# Remarks on Obstructions of the Intestines

**Published:** 1825-10

**Authors:** Joseph Morley

**Affiliations:** Surgeon.


### Art. IV.—*Remarks on Obstructions of the Intestines.* By Joseph Morley, Esq. Surgeon.

The following observations upon obstructions of the ilium, particularly that which is occasioned by an introsusception of that intestine, may be useful to the young practitioner, and I trust they will not be entirely devoid of interest to the more experienced. I am of opinion in strangulated hernia (which involves this intestine), if a surgical operation were performed shortly after the surgeon had discovered the cause of the alarming symptoms, nine times in ten human existence would be preserved. If the operation is performed by a skilful surgeon, there is scarcely any danger with respect to the operation itself. The fatality of the disease is occasioned by procrastination, and an obstinate perseverance in means which accelerate the fatal termination of this dreadful and painful disease.

I need not here enumerate the symptoms which lead to the discovery of strangulated hernia; but I will say it is the imperative duty of every medical attendant, in cases of obstinate constipation of the intestines, to ascertain, in the first instance, whether such constipation is occasioned by strangulated hernia, which may be easily done by an examination; and, if such is the case, and it cannot be reduced by gentle means of *short duration*, immediately to recommend the operation, and in as decided terms as possible. An introsusception of the ilium cannot be discovered by any external examination of the body; therefore it is much more difficult to discover this disease than the other, though the same intestine is involved in each. If the obstruction is not speedily removed, inflammation of the bowels must ensue; and, before medical assistance is obtained, this generally, more or less, has taken place. If the effect is mistaken for the cause, nothing else than death, in all probability, will take place; for the more likely way of removing the obstruction is by mechanical means: but what medical man would recommend such means, when at the same time his mind was impressed with the idea that inflammation alone was the whole and sole cause of the obstruction? My mind will never be so impressed, because I am of a firm opinion that inflammation is merely the effect of a primary agent: "remove the cause, and the effect will cease."

It requires very minute attention, almost amounting to an impossibility, to discover at first view (if unaccompanied with hernia,) from what cause constipation proceeds: it may be from hardened fæces or introsusception, or both; but, fortunately, in the first instance, enemas, accompanied with bleeding, the usual methods resorted to, are proper in either case. If a copious evacuation is obtained by such means, and even hardened fæces are expelled, we must not be deceived; for, if there is an obstruction in the ilium, the enema will empty the larger intestines *below the stricture*, but the cause will still remain. If, after such an evacuation, pain should return in the umbilical region, attended with a quickened pulse, anxiety in the countenance, and flushed cheeks, I would trust no longer to the usual routine of practice, but draw the conclusion the disease was in the ilium, and immediately proceed to mechanical treatment. Procrastination at this period, as in strangulated hernia, is dangerous.

I am strongly of an opinion that the reason why mercury, given in a crude state, should be out of fashion in our time, has arisen from its having been given at too late a period; and, when death has taken place under its exhibition, the fatal termination has been imputed to the remedy, instead of the disease. Mercury in its crude state will soon pass through the whole of the intestinal canal, unless there is a serious obstruction. If it meets with this obstruction, there is a probability that it will be overcome by its specific gravity. Not less than a pound should be given. If it should be retained days in the crude state, its active principle will remain dormant, and therefore it will do no harm. The retention of it proves the obstinacy of the disease, and, should it prove fatal, the mercury would have no more to do in hastening that event, than if it never had been employed.

Mercury is the first mechanical power that I would recommend in introsusception of the ilium, and *at an early period;* the second, distention, by injecting per anum to the extent of three or four quarts of gruel, by the aid of Read's patent syringe: (no surgeon should be without one.) As the mechanical power of mercury acts by its specific gravity, the same power of the injection acts by distention; therefore there must be such a quantity thrown up as will reach the obstruction. A mild purgative enema should always be used in the first instance, to empty the lower bowels of their contents.

*Wellingore; August 2d,* 1825.

